# Learning Active Depth Sensing for UAV Navigation: Reliability Supervision and Staged Adaptation

**DOI:** 10.3390/s26216732

**Published:** 2026-10-24

**Authors:** Weiqi Gai, Guodong Zhao, Jiachi Ji, Zhijun Meng

**Affiliations:** School of Aeronautic Science and Engineering, Beihang University, Beijing 100191, China

**Keywords:** active depth sensing, UAV navigation, differentiable sensing, depth reliability, camera control, flight adaptation

## Abstract

Adaptive depth acquisition changes the observations used for UAV navigation. We present a staged framework that connects differentiable depth-reliability supervision to flight performance. An active-stereo surrogate supplies power, exposure, and gain targets for the least reliable image patches. Student-rollout relabeling extends supervision to learned-acquisition states, and independent visual stems preserve the camera mapping during flight adaptation. In the primary simulated aperture-crossing benchmark (60-step cap), success reaches 75.1% versus 66.7% for nominal fixed acquisition, while valid-depth fill rises from 72.1% to 97.5%. Under a separate ablation protocol (5 s timeout; 120-step cap), removing relabeling and retraining lowers success from 77.89% to 70.78%; sharing the visual stem lowers it to 74.30%. The frozen policy retains 75.33%–79.67% success across four partial-observation layouts. A bounded-depth-bias test yields 70.00% success at 0.10 m and 37.00% at 0.30 m, compared with its 74.67% zero-bias control. Supplementary D455 captures show a coverage gain after camera adaptation, and a separate PX4 hardware-in-the-loop test completes 44/60 crossings with synthetic depth. The results demonstrate navigation gains from the complete differentiable-sensing and flight-adaptation framework while quantifying its sensitivity to incorrect ranges.

## 1. Introduction

Autonomous unmanned aerial vehicles (UAVs) need local geometry to avoid obstacles and traverse confined spaces. Compact depth cameras support these tasks, but measurement availability depends on acquisition conditions. For aperture crossing, the relevant sensing requirement is local: depth must reveal the boundary needed for the next motion decision, even when most background pixels remain valid.

Active stereo makes this requirement controllable: projector power, exposure, and gain affect correspondence through material, illumination, range, and motion [1,2]. Perception-aware flight changes trajectories or speed to preserve observations [3,4,5]; acquisition control changes the measurement at a given pose [6,7,8]. We study the latter interface for depth-based aperture crossing.

**Figure 1:**
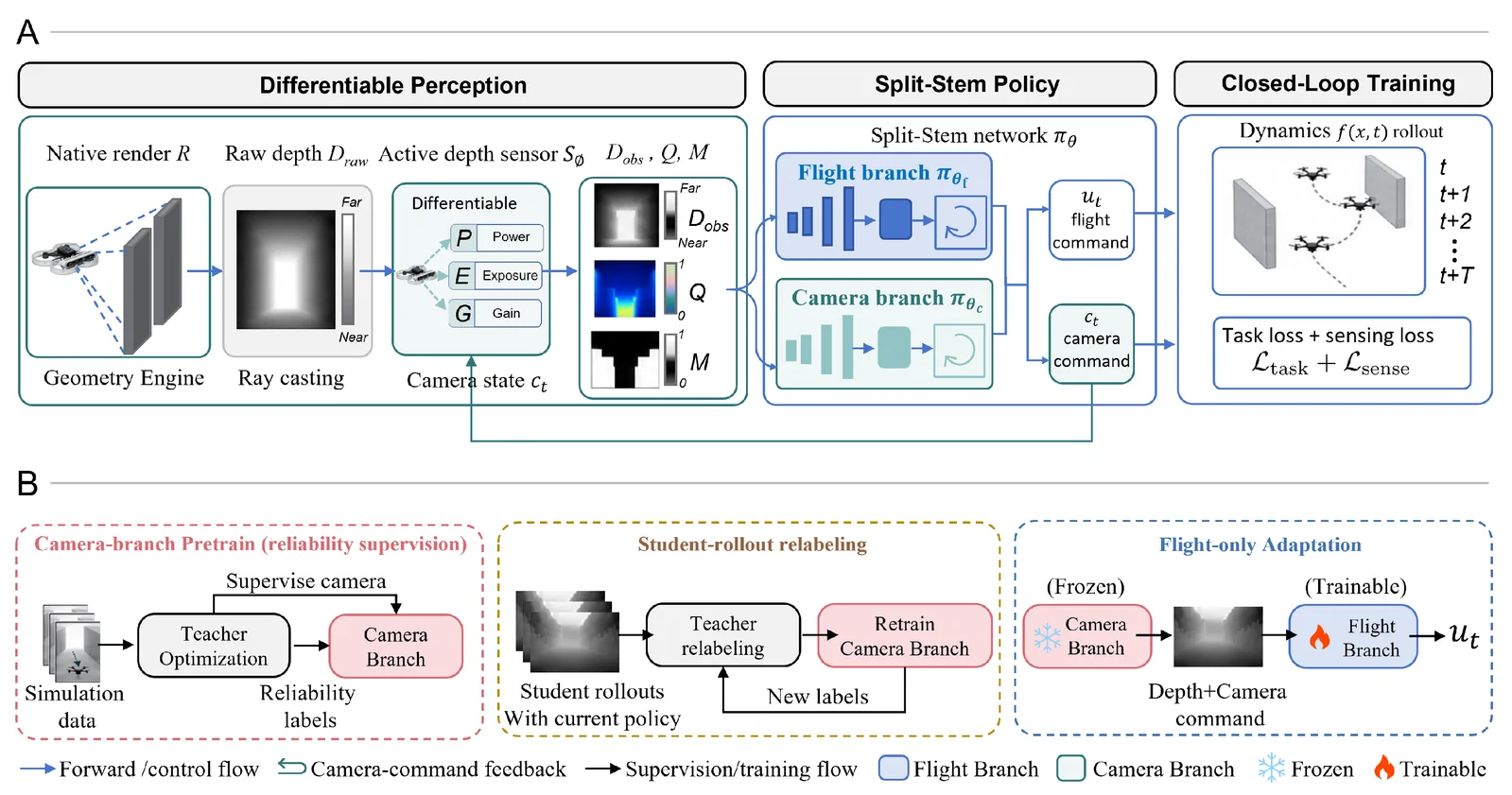
Staged sensing and flight learning. (**A**) Acquisition-dependent depth feeds independent camera and flight branches. (**B**) Reliability teaching and camera fitting are followed by student-state relabeling/retraining and flight adaptation with the complete camera mapping frozen. Quality maps are sensor auxiliaries; only observed depth enters the policy. Blue and teal identify the flight and camera branches, respectively. Blue solid arrows indicate forward data and control flow, and the teal feedback arrow indicates camera-command feedback for subsequent acquisition. Blue dashed rays illustrate geometric depth rendering. Arrows in panel (**B**) indicate supervision and training flow; colored dashed boxes distinguish training stages. Snowflake and flame symbols denote frozen and trainable parameters, respectively.

Learning acquisition for navigation involves two distribution shifts. Camera decisions change the observations received by the camera controller itself, and flight must learn to use the resulting depth stream. Updating a shared encoder during flight adaptation can additionally change the camera mapping. Figure 1 addresses these dependencies by teaching local depth reliability, querying the teacher on student-induced states, and adapting flight while freezing an independent camera branch.

The contribution is a learning interface that makes differentiable acquisition useful for downstream control. Its three components provide reliability-based register supervision, camera learning on student-induced states, and flight adaptation with a preserved camera mapping. The primary navigation comparisons establish the complete-system gain; separate ablations test the latter two design choices. Added occlusion and depth-bias tests examine the frozen policy’s operating range, while physical D455 acquisition and PX4 simulation-in-hardware (SIH) test the sensing and flight-controller interfaces separately.

## 2. Related Work

We organize prior work by sensor response, navigation, and learning under adaptive observations: the three interfaces connected by the proposed method.

### 2.1. Depth Measurement and Adaptive Camera Acquisition

RealSense metrology [1] and studies of pattern projection and gain in active stereo [2] motivate our response factors. Exposure optimization for visual odometry links a photometric proxy to downstream localization [6]. We similarly distinguish the sensing objective from task performance, but control projector power, exposure, and gain to regulate depth availability during recurrent flight.

### 2.2. Depth-Based and Perception-Aware UAV Navigation

Depth-based systems combine route following and depth thresholds [9], actuated sensing for landing and avoidance [10], or directional constraints under depth dropout [11]. Active perception [12,13,14] also motivates viewpoint, trajectory, and speed control [3,4,5,15,16]. Agile-flight systems further demonstrate tight perception–action coupling [17,18,19]. Register adaptation complements these approaches by changing the depth acquired at a given pose.

### 2.3. Differentiable Sensing and Learning with Adaptive Observations

Differentiable simulation supports gradient-based flight learning [20,21,22,23] and differentiable model-predictive control [24]. Embodied simulation [25,26,27,28], image-system optimization, and sensor transfer [29,30,31,32,33] connect observation formation to learning. Neural Camera Simulators and DiffPhysCam expose controllable image transformations and camera response [34,35]; our surrogate follows their modular separation of geometry and acquisition.

TaCOS and JOCA optimize camera design or adaptive camera control jointly with perception [7,8]. We instead keep hardware fixed and connect local depth-reliability teaching to recurrent flight adaptation. DAgger supplies the learner-state querying principle [36]: here the teacher labels acquisition registers while the student retains flight control. The ablation tests this relabeling together with camera retraining, rather than proposing a new aggregation algorithm.

## 3. Materials and Methods

The teacher is a finite-step optimizer that supplies camera-register targets through a differentiable sensing surrogate; the student is the recurrent camera controller. Training proceeds from an initial teacher collection to camera pretraining, student-rollout relabeling and camera retraining, then flight adaptation with the entire camera mapping frozen. Algorithm 1 summarizes the sequence.
**Algorithm 1** Reliability Teaching, Camera Learning, and Flight Adaptation**Require:** fixed flight policy, sensing regimes, teacher objective (21) 1:Collect $D_{0}$ under the initial acquisition policy.2:At each collected state, optimize a camera target with geometry and motion fixed.3:Fit only $\Theta_{\mathrm{cam}}$ on $D_{0}$ using (24).4:Roll out the camera-pretrained student with its own flight and acquisition actions.5:Query the same teacher at visited states to form $D_{\mathrm{dag}}$.6:Retrain $\Theta_{\mathrm{cam}}$ on $D_{\mathrm{dag}}$.7:Freeze the complete camera mapping and detach its recurrent path.8:Adapt $\Theta_{f}=\Theta \setminus \Theta_{\mathrm{cam}}$ under learned acquisition using (27).9:**return** final split-stem policy


### 3.1. Problem Formulation and Closed-Loop Acquisition

Let $x_{t}$ contain pose, velocity, attitude, and collision margin, and let E specify the scene geometry and sensing regime $\rho_{t}$. Scene denotes the spatial layout; regime denotes its glare, dark-material, or specular acquisition response. Normalized projector power, exposure, and gain form the camera state

(1) $$c_{t}={\left[P_{t},E_{t},G_{t}\right]}^{⊤}\in {\left[0,1\right]}^{3},$$

The closed loop is

(2) $$\begin{array}{cc} D_{t}^{\mathrm{raw}} & =R(x_{t},E), \end{array}$$

(3) $$\begin{array}{cc} \left(D_{t}^{\mathrm{obs}},Q_{t}^{\mathrm{obs}}\right) & =S_{\phi }\left(D_{t}^{\mathrm{raw}},c_{t},\rho_{t},x_{t}\right), \end{array}$$

(4) $$\begin{array}{cc} s_{t} & =\psi_{\mathrm{flight}}\left(x_{t},x_{\mathrm{goal}},c_{t}\right), \end{array}$$

(5) $$\begin{array}{cc} m_{t} & =\psi_{\mathrm{cam}}\left(x_{t},c_{t}\right), \end{array}$$

(6) $$\left(u_{t},c_{t+1}^{★},h_{t+1},q_{t+1}\right)=\Pi_{\theta }\left(D_{t}^{\mathrm{obs}},s_{t},c_{t},m_{t},h_{t},q_{t}\right),$$

(7) $$\begin{array}{cc} c_{t+1} & =\alpha c_{t}+\left(1-\alpha \right)c_{t+1}^{★},\;\alpha =0.7, \end{array}$$

(8) $$\begin{array}{cc} x_{t+1} & =f_{\mathrm{dyn}}\left(x_{t},a_{t-2},\Delta t_{t}\right). \end{array}$$

The renderer R produces ideal depth and $S_{\phi }$ produces observed depth and auxiliary quality. Only observed depth enters the policy. Flight inputs $s_{t}$ contain velocity, goal-directed reference, attitude, safety margin, and current registers rescaled to [−1,1]; camera-state feedback is included in all reported experiments. Camera motion input $m_{t}$ excludes the goal vector. Hidden states $h_{t}$ and $q_{t}$ serve the two branches. The acceleration output $u_{t}$ is clipped to $\pm a_{\max}$, rotated to the world frame to obtain $a_{t}$, and applied after two control steps. Primary and ablation runs use simulator state; SIH uses PX4 estimated state (Section 4.5).

(9) $$\Delta t_{t}=\Delta t_{\mathrm{base},t}+\kappa_{\tau }\tau \left({\langle E_{t}\rangle }_{\mathrm{batch}}\right),$$

Here $\kappa_{\tau }=0.01$ and the base interval has mean 1/15 s and standard deviation 0.1/15 s. The batch-mean exposure term is detached from gradients to share one integration interval across vectorized rollouts. With τ(E)=0.25+2.75E, exposure adds 2.5–30 ms: the nominal total is 69.17–96.67 ms before jitter, and the maximum individual-versus-mean discrepancy is 27.5 ms. Evaluation uses batch size one, making batch-mean and per-state timing identical. The training integrator is held fixed across comparisons. These parameters define the acquisition timing of the simulation model.

### 3.2. Differentiable Active-Stereo Response Model

**Figure 2:**
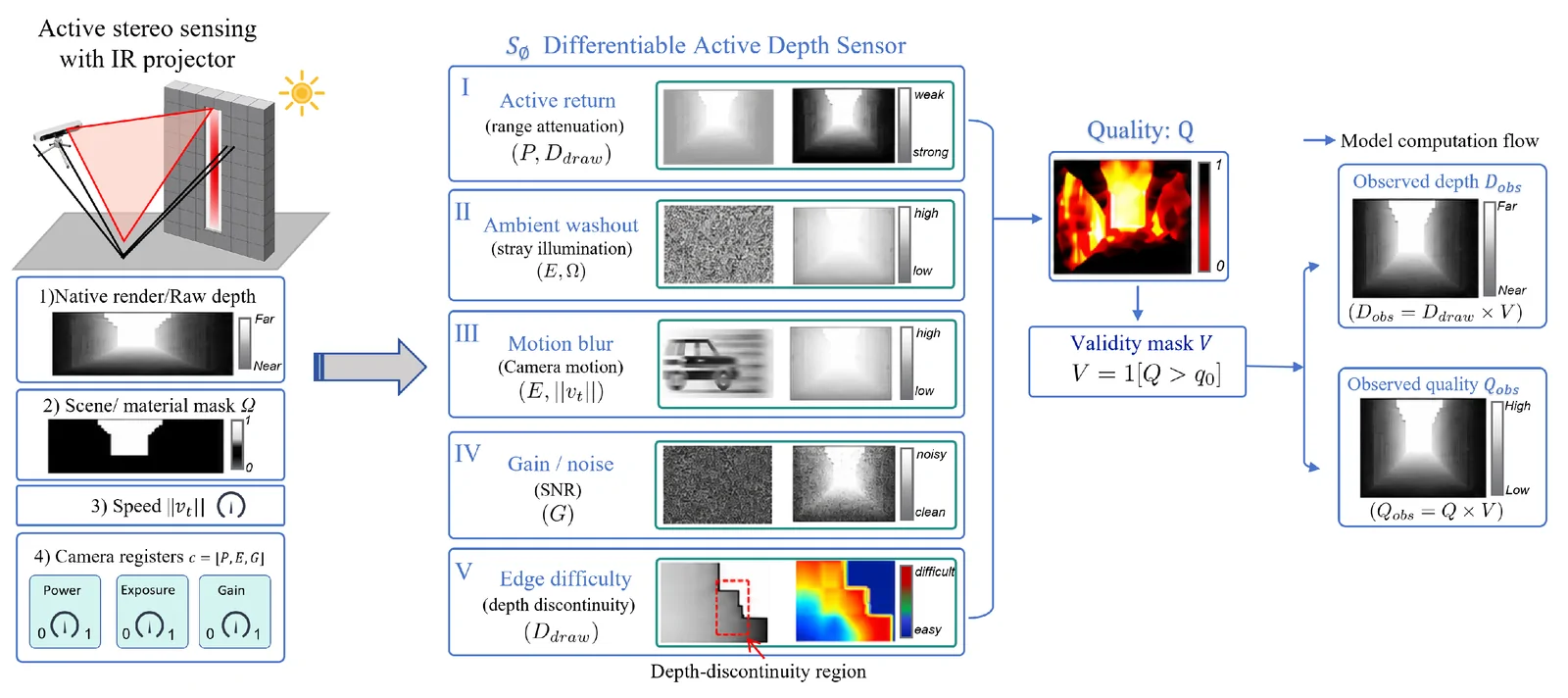
Conceptual active-stereo response model. Rendered depth, localized sensing hazards, motion, and power/exposure/gain settings determine acquisition quality *Q* through five illustrated response factors: (I) active return, (II) ambient washout, (III) motion blur, (IV) gain-dependent noise, and (V) depth-edge difficulty. Hard validity *V* gates depth and quality to produce $D^{\mathrm{obs}}$ and the auxiliary $Q^{\mathrm{obs}}$; only observed depth enters the policy. The image examples illustrate response mechanisms, rather than intermediate images generated by the surrogate. Blue arrows indicate model computation flow, and the red dashed box highlights a depth-discontinuity region. Colored maps schematically illustrate spatial variation in sensing response and quality. Time and pixel indices are omitted.

The teacher compares acquisition settings at a fixed state. Following modular camera simulation [35], geometric rendering supplies ideal depth $Z_{t}=D_{t}^{\mathrm{raw}}$ and the acquisition response predicts its availability. Figure 2 groups projected return, washout, blur, noise, and edge difficulty; the surrogate models dropout rather than photometric corruption or biased retained ranges.

For camera position $p_{t}^{E}$ and orientation columns $\left(r_{x,t},r_{y,t},r_{z,t}\right)$, the pixel ray is

(10) $$\begin{array}{cc} \eta_{i} & =\left(2\frac{i+1/2}{H}-1\right)f_{y}-\epsilon_{r},\;f_{y}=f_{x}\frac{H}{W},\\ \xi_{j} & =\left(2\frac{j+1/2}{W}-1\right)f_{x}-\epsilon_{r},\\ d_{tij}\; & =r_{x,t}-\eta_{i}r_{z,t}-\xi_{j}r_{y,t}, \end{array}$$

Here $f_{x}=\tan\left({\mathrm{FOV}}_{x}/2\right)$ and $\epsilon_{r}$ is a numerical offset. Ideal depth is the smallest positive ray-intersection parameter. Clipping to $\bar{Z}=\mathrm{clip}\left(Z,z_{\min},z_{\max}\right)$ gives the neighborhood discontinuity score

(11) $$\begin{array}{cc} Z_{tij}^{+} & =\max_{\left(m,n\right)\in N_{ij}}{\bar{Z}}_{tmn},\;Z_{tij}^{-}=\min_{\left(m,n\right)\in N_{ij}}{\bar{Z}}_{tmn},\\ \delta_{tij} & =\mathrm{clip}\left(\frac{Z_{tij}^{+}-Z_{tij}^{-}}{{\bar{Z}}_{tij}+\epsilon_{z}},0,1\right). \end{array}$$

The 3×3 neighborhood $N_{ij}$ approximates correspondence difficulty near depth boundaries. A projected hazard mask $\Omega_{tij}=m_{\rho_{t}}\left({\bar{Z}}_{tij},x_{t},E\right)\in \left[0,1\right]$ localizes illumination or material degradation. Geometry, pose, and this mask are held fixed when differentiating camera registers. For clipped commands $p_{t},e_{t},g_{t}\in \left[0,1\right]$, the exposure and gain mappings are

(12) $$\begin{array}{cc} \tau (E_{t}) & =t_{\min}+t_{\mathrm{span}}e_{t}, \end{array}$$

(13) $$\begin{array}{cc} \Gamma (G_{t}) & =\Gamma_{0}+\Gamma_{s}\frac{{\left(g_{t}+\epsilon_{g}\right)}^{\gamma }-\epsilon_{g}^{\gamma }}{{\left(1+\epsilon_{g}\right)}^{\gamma }-\epsilon_{g}^{\gamma }}. \end{array}$$

Active and passive returns are

(14) $$\begin{array}{cc} S_{tij}^{\mathrm{act}} & =\frac{k_{a}p_{t}\tau \left(E_{t}\right)}{{\bar{Z}}_{tij}^{2}+c_{d}},\\ S_{t}^{\mathrm{pass}} & =k_{p}\tau \left(E_{t}\right)\sqrt{\Gamma (G_{t})}, \end{array}$$

(15) $$\begin{array}{cc} S_{tij} & =S_{tij}^{\mathrm{act}}+S_{t}^{\mathrm{pass}}. \end{array}$$

The inverse-square term models projected-pattern attenuation; the passive term captures ambient or texture-assisted correspondence. Signal, washout, motion, edge, and range factors yield

(16) $$\begin{array}{cc} I_{tij}^{\mathrm{amb}} & =a_{0}+a_{1}\Omega_{tij},\;R_{tij}={\left[{\bar{Z}}_{tij}/z_{\max}-r_{0}\right]}_{+},\\ B_{t}^{\mathrm{mot}} & =\mathrm{clip}(k_{m}∥v_{t}{∥}_{2}\tau \left(E_{t}\right),0,b_{\max}),\\ W_{tij}^{\mathrm{out}} & =\frac{I_{tij}^{\mathrm{amb}}\tau \left(E_{t}\right)}{S_{tij}^{\mathrm{act}}+c_{w}},\;N_{tij}^{\mathrm{shot}}=\frac{n_{0}\left(n_{1}+n_{2}\Gamma \left(G_{t}\right)\right)}{S_{tij}+c_{n}},\\ \chi_{tij} & =\frac{S_{tij}}{b_{0}+b_{1}I_{tij}^{\mathrm{amb}}+b_{2}N_{tij}^{\mathrm{shot}}+b_{3}B_{t}^{\mathrm{mot}}\left(b_{4}+\delta_{tij}\right)},\\ Q_{tij}^{0} & =\sigma \left(\beta_{s}\chi_{tij}-\beta_{w}W_{tij}^{\mathrm{out}}-\beta_{e}\delta_{tij}-\beta_{r}R_{tij}\right). \end{array}$$

The terms $I^{\mathrm{amb}}$, $W^{\mathrm{out}}$, $N^{\mathrm{shot}}$, and χ denote ambient infrared level, washout, noise proxy, and signal-to-noise proxy. Their coefficients encode qualitative active-stereo response regimes. Regime-specific penalties $A_{\rho }$ and bonuses $B_{\rho }$ adjust quality inside the hazard mask:

(17) $$\begin{array}{cc} C_{\rho ,t} & =B_{\rho_{t}}\left(p_{t},e_{t},g_{t}\right)-A_{\rho_{t}}\left(p_{t},e_{t},g_{t}\right),\\ Q_{tij} & =\mathrm{clip}\left(Q_{tij}^{0}+\Omega_{tij}C_{\rho ,t},0,1\right). \end{array}$$

Glare favors high power with low exposure/gain; specularity penalizes excessive active return; dark material rewards recovery of weak returns while retaining blur/noise costs. The resulting quality score defines soft and hard validity:

(18) $$\begin{array}{cc} {\tilde{V}}_{tij} & =\sigma \left(\left(Q_{tij}-q_{0}\right)/s_{v}\right),\;V_{tij}=I\left[Q_{tij}>q_{0}\right],\\ D_{tij}^{\mathrm{obs}} & ={\bar{Z}}_{tij}V_{tij},\;Q_{tij}^{\mathrm{obs}}=Q_{tij}V_{tij}. \end{array}$$

We use $q_{0}=0.42$ and $s_{v}=0.055$. Valid pixels retain clipped depth; specular blooming is approximated by dropout, not multipath-induced false surfaces. Here *Q* is a reliability score and $\tilde{V}$ is its smooth validity gate. The teacher uses soft validity; forward observations use hard validity, with straight-through gate $\mathrm{sg}\left[V-\tilde{V}\right]+\tilde{V}$ when needed. Evaluation reports full-image fill

(19) $$F_{t}=\frac{1}{HW}\sum_{i=0}^{H-1}\sum_{j=0}^{W-1}V_{tij}.$$

### 3.3. Reliability-Based Camera Teaching

Image-wide fill can hide a poorly observed aperture boundary. The teacher, therefore, targets the least reliable patches at each fixed state, optimizing a bounded target $\bar{c}=\mathrm{clip}\left(\sigma \left(\eta \right),\epsilon ,1-\epsilon \right)$ with ϵ=0.001. A 6×8 adaptive-pooling grid and lower-tail fraction β define

(20) $$\begin{array}{cc} p(\bar{c}) & =\mathrm{vec}\;\left({\mathrm{Pool}}_{\mathrm{avg}}^{R_{p}\times C_{p}}\left[\tilde{V}\left(\bar{c}\right)\right]\right),\\ K & =\left\lceil \beta R_{p}C_{p}\right\rceil ,\\ r_{\mathrm{fill}}\left(\bar{c}\right) & =\frac{1}{K}\sum_{i\in B_{K}\left(p\right)}p_{i}, \end{array}$$

The set $B_{K}$ contains the *K* smallest patch means. This piecewise-smooth statistic emphasizes weak regions without explicitly identifying the aperture. The teacher balances reliability, register variation, and acquisition costs:

(21) $$\begin{array}{cc} {\bar{c}}_{t+1} & =\arg\min_{\bar{c}\in {\left[\epsilon ,1-\epsilon \right]}^{3}}L_{\mathrm{teach}}\left(\bar{c}\right),\\ L_{\mathrm{teach}}= & \lambda_{f}{\left[r_{\min}-r_{\mathrm{fill}}\left(\bar{c}\right)\right]}_{+}^{2}+\frac{\lambda_{s}}{3}{∥\bar{c}-c_{t}∥}_{2}^{2}\\ \; & +\lambda_{P}{\left[\bar{P}-P_{0}\right]}_{+}^{2}+\lambda_{b}{∥v_{t}∥}_{2}^{2}\tau {\left(\bar{E}\right)}^{2}\\ \; & +\lambda_{G}{\bar{G}}^{2}+\frac{\lambda_{0}}{3}\omega \left(\bar{c}\right){∥\bar{c}-c_{\mathrm{nom}}∥}_{2}^{2}. \end{array}$$

The terms penalize insufficient reliability, register changes, excess power, motion-dependent exposure blur, and gain. Nominal recovery is weighted by

(22) $$\omega \left(\bar{c}\right)=\mathrm{sg}\;\left[\mathrm{clip}\;\left(\frac{r_{\mathrm{fill}}\left(\bar{c}\right)-r_{\min}}{m_{\mathrm{fill}}},0,1\right)\right],$$

Here sg stops gradients through the recovery gate. Logits start at $\mathrm{logit}(\mathrm{clip}\left(c_{t},\epsilon ,1-\epsilon \right))$; Adam returns a finite-step label rather than an exact minimizer. Primary teaching uses 120 steps at learning rate 0.08. Numerical weights and ablation budgets are given in Table 1 and Table 2. Teaching is offline and adds no iterative optimization to deployed policy inference.

### 3.4. Learning Camera Control on Student-Induced States

**Figure 3:**
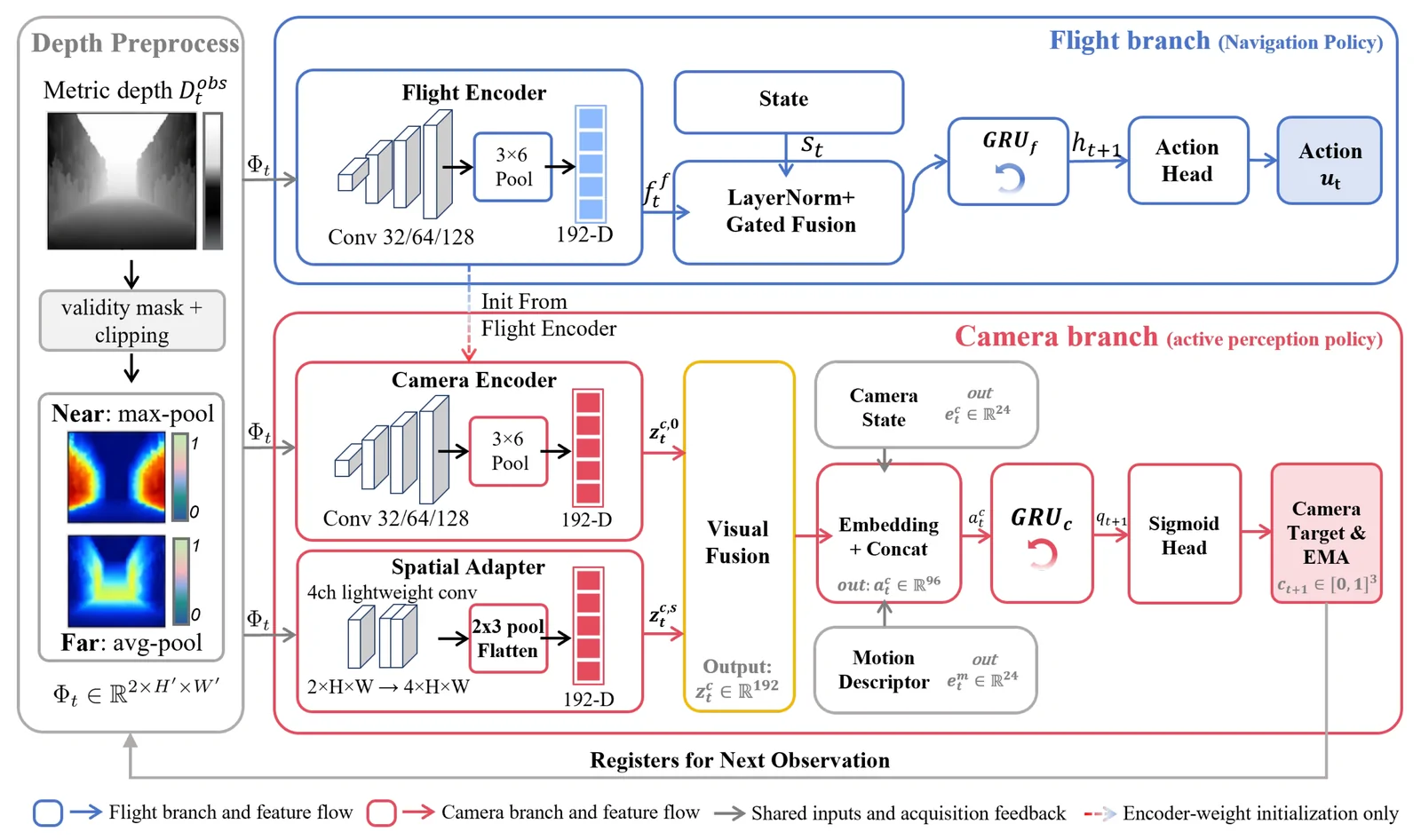
Split-stem recurrent policy. Depth preprocessing supplies near-obstacle and far-opening channels to independent flight and camera encoders. The flight branch fuses visual and vehicle-state features to output local acceleration $u_{t}$. The camera branch combines global and spatial depth features with register and motion embeddings to predict an absolute camera target, followed by exponential moving average (EMA) smoothing. The dashed inter-stem arrow denotes initialization from the flight stem; subsequent parameters are independent. The bottom feedback arrow summarizes acquisition of the next depth frame through the sensor model. Blue and red identify the flight and camera branches and their feature flow, respectively. Gray arrows indicate shared inputs and acquisition feedback. Curved arrows within the gated recurrent units (GRUs) denote recurrent state updates.

The camera and flight branches have independent visual stems (Figure 3) but receive the same acquired depth. A camera collection contains observation/register/motion inputs $o_{b,t}^{c}$ and teacher targets

(23) $$D={\left\{\left(o_{b,t}^{c},{\bar{c}}_{b,t+1}\right)\right\}}_{b,t}.$$

Camera fitting unrolls its recurrent state over *B* sequences of *T* steps and minimizes

(24) $$\begin{array}{cc} L_{\sup}= & \frac{1}{3BT}\sum_{b=1}^{B}\sum_{t=0}^{T-1}\rho_{\delta }\left(c_{b,t+1}^{★}-{\bar{c}}_{b,t+1}\right)\\ \; & +\lambda_{\Delta c}\frac{1}{3B(T-1)}\sum_{b=1}^{B}\sum_{t=1}^{T-1}{∥c_{b,t+1}^{★}-c_{b,t}^{★}∥}_{2}^{2}, \end{array}$$

Here $\rho_{\delta }$ sums component-wise SmoothL1 losses; division by three averages register channels. Camera fitting uses δ=1 and $\lambda_{\Delta c}=0$; temporal register regularization is, therefore, disabled during fitting. Only camera parameters $\Theta_{\mathrm{cam}}$ are updated. Because learned acquisition changes subsequent observations, pretraining on $D_{0}$ induces a distribution shift at deployment. We query the same teacher on student rollouts and retrain on

(25) $$D_{\mathrm{dag}}=\left\{\left(\xi_{t},{\bar{c}}_{t+1}\right)\;|\;\xi_{t}∼\mu \left(\theta_{\mathrm{pre}}\right),\;{\bar{c}}_{t+1}=\arg\min_{\bar{c}}L_{\mathrm{teach}}\right\}.$$

The distribution $\mu (\theta_{\mathrm{pre}})$ includes the student’s flight and acquisition history; $\xi_{t}$ denotes the visited state. The student retains flight control and executes its own smoothed camera targets. One relabeling/retraining round is used; DAgger in the experiment labels denotes this combined stage. Collection sizes are specified separately from evaluation horizons in Table 3.

### 3.5. Flight Adaptation Under Learned Acquisition

The final stage learns navigation under the new depth stream while preserving the camera mapping. Frozen parameters do not imply constant registers: camera actions remain responsive to observations. The camera output is detached from flight gradients,

(26) $$c_{t+1}^{★}=\mathrm{sg}\;\left[\Pi_{\theta_{\mathrm{cam}}}^{c}\left(D_{t}^{\mathrm{obs}},c_{t},m_{t},q_{t}\right)\right],\;\theta_{\mathrm{cam}}\;\mathrm{fixed},$$

and flight minimizes

(27) $$\begin{array}{cc} L_{\mathrm{flight}}= & \lambda_{v}{\bar{L}}_{v}+\lambda_{a}{\bar{L}}_{\mathrm{acc}}\\ \; & +\lambda_{j}{\bar{L}}_{\mathrm{jerk}}+\lambda_{c}{\bar{L}}_{\mathrm{collide}},\\ \theta^{★}= & \arg\min_{\Theta_{f}}E_{\rho ,x_{0}}L_{\mathrm{flight}}. \end{array}$$

Overbars average valid time indices and the batch. Velocity tracking uses a $W_{v}=12$ step window and the same SmoothL1 transition δ=1, with a goal-directed reference,

(28) $$L_{v}\left(t\right)=\rho_{\delta }\;\left({∥\frac{1}{W_{v}}\sum_{i=0}^{W_{v}-1}v_{t-i}-v_{t}^{\mathrm{ref}}∥}_{2}\right),$$

(29) $$L_{\mathrm{acc}}\left(t\right)={∥a_{t}∥}_{2}^{2},\;L_{\mathrm{jerk}}\left(t\right)={∥15\left(a_{t}-a_{t-1}\right)∥}_{2}^{2}.$$

The jerk scale uses a nominal 15 Hz. Clearance is queried at ten extrapolated positions $p_{t}+v_{t}k/\left(9\cdot 15\right)$, k=0,…,9, using a spherical body radius of 0.12 m and zero additional training margin. These finite lookahead queries approximate continuous swept-volume checking. With clearance $d_{t,k}$, the barrier is

(30) $$L_{\mathrm{collide}}\left(t\right)=\frac{1}{K_{o}}\sum_{k=1}^{K_{o}}\zeta_{t,k}\;\mathrm{softplus}\left(-32\max\left(d_{t,k},-3\right)\right),$$

Here $K_{o}=9$: the barrier averages the nine forward samples k=1,…,9, while k=0 supplies the initial clearance for differencing. The detached factor $\zeta_{t,k}=\mathrm{sg}\left[\max\left(1,135\left(d_{t,k-1}-d_{t,k}\right)\right)\right]$ upweights decreasing clearance along this lookahead, not across rollout time. Auxiliary avoidance and camera losses have zero weight during flight adaptation.

### 3.6. Policy Implementation

Observed depth is encoded into near and far channels. Define $V^{d}=I\left[D^{\mathrm{obs}}\geq z_{\min}\right]$ and let $\bar{D}$ equal clipped valid depth, with $z_{\max}$ substituted at invalid pixels. Then

(31) $$\begin{array}{cc} \phi_{t}^{\mathrm{near}} & ={\mathrm{Pool}}_{\max}^{H_{d}\times W_{d}}\left[V_{t}^{d}⊙\mathrm{clip}\left(\frac{1/{\bar{D}}_{t}-1/z_{\max}}{1/z_{\min}-1/z_{\max}},0,1\right)\right],\\ \phi_{t}^{\mathrm{far}} & ={\mathrm{Pool}}_{\mathrm{avg}}^{H_{d}\times W_{d}}\left[V_{t}^{d}⊙\mathrm{clip}\left(\frac{{\bar{D}}_{t}-z_{\min}}{z_{\max}-z_{\min}},0,1\right)\right],\\ \Phi_{t} & =2[\phi_{t}^{\mathrm{near}},\phi_{t}^{\mathrm{far}}]-1. \end{array}$$

The 36×48 near channel uses inverse depth and max pooling; the far channel uses metric depth and average pooling. Both suppress invalid pixels. Each independent visual stem has 32/64/128 convolutional channels, stride-2 downsampling in the second block, 3×6 pooling, and a 192-dimensional projection. The flight branch gates layer-normalized visual and state features, updates a GRU, applies a 0.1-scaled residual MLP and layer normalization, and predicts acceleration. LeakyReLU uses slope 0.05.

The camera stem is initialized from the flight stem but shares no parameters thereafter. A 4-channel spatial adapter with 2×3 pooling and a residual image adapter contribute with scales 0.35 and 0.25, respectively. Current registers $2c_{t}-1$ and the motion descriptor

(32) $$r_{t}^{\mathrm{up}}=R_{t}e_{3},\;m_{t}=\left[\mathrm{clip}\left(v_{t}^{\mathrm{local}}/v_{\max},-2,2\right),r_{t}^{\mathrm{up}}\right]\in R^{6},$$

are each embedded into 24 dimensions and fused with image features. Here $e_{3}={\left[0,0,1\right]}^{⊤}$; the up vector is in the world frame. The fused camera feature $a_{t}^{c}$ feeds a 96-dimensional GRU; with φ denoting LeakyReLU and $W_{o}$ the affine output map, it predicts

(33) $$q_{t+1}=\mathrm{LN}\left({\mathrm{GRU}}_{c}\left(a_{t}^{c},q_{t}\right)\right),\;c_{t+1}^{★}=\sigma \left(W_{o}\varphi \left(q_{t+1}\right)\right).$$

All camera visual modules, state/motion embeddings, fusion layers, recurrent units, output heads, and normalization layers belong to $\Theta_{\mathrm{cam}}$. They are trained during camera fitting and frozen during flight adaptation. The sigmoid yields absolute normalized registers; Equation (7) applies smoothing outside the network.

## 4. Experimental Setup

Experiments use a custom PyTorch/CUDA simulator, available at https://github.com/Gaiwqqq/ActiveDepthSensingForUAV (accessed on 21 October 2026). The archived simulation environment specifies Python 3.9.23, PyTorch 2.1.0, CUDA 12.1, NumPy 1.26.4, and pandas 2.1.3. It models vehicle dynamics, ray-based depth, and obstacle geometry; the acquisition surrogate converts ideal ranges into register-dependent observations. This controlled aperture-crossing study evaluates complete-policy navigation, training design, and robustness, with separate physical-camera and SIH experiments.

### 4.1. Benchmark and Training Configuration

**Figure 4:**
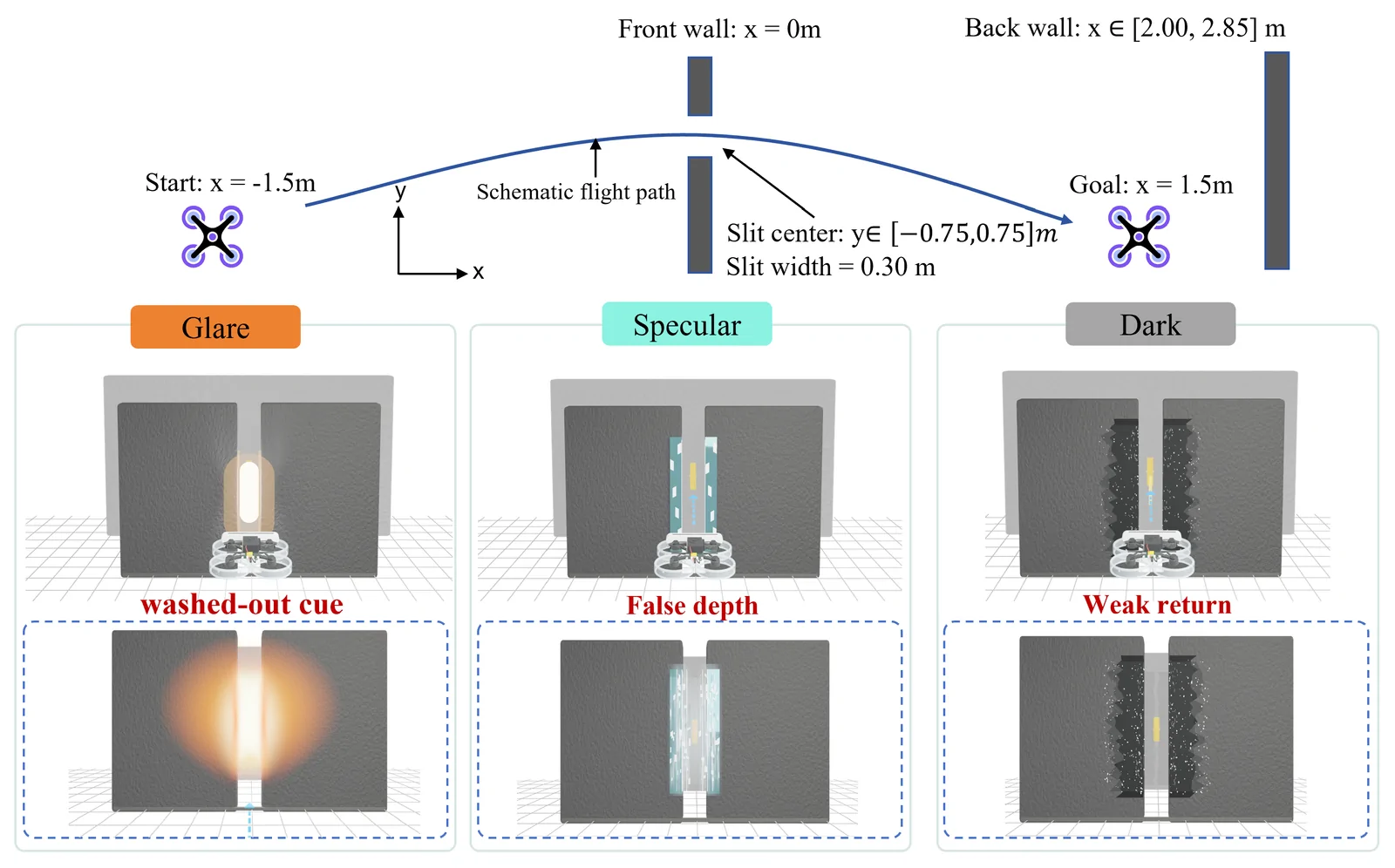
Conceptual evaluation scenes. Top: aperture-crossing geometry in the local scene frame, with coordinates in meters; the blue curve is a schematic path, not an evaluated trajectory, and the drawing is not to scale. Bottom, left to right: glare, specular, and dark-material sensing regimes, each with an approach view and a closer view of the affected region. The illustrations depict physical mechanisms motivating the surrogate; the implemented response models their effect on depth availability, with valid pixels retaining rendered depth. These are conceptual illustrations, not simulator screenshots or physical flight experiments. Geometry and randomization are shared across regimes. Colored headers identify the sensing regimes, and blue dashed boxes identify close-up views. The “False depth” label illustrates possible specular range errors; the base surrogate represents specular degradation through dropout rather than biased ranges. The separate test in Section 5.4 injects range bias explicitly.

Table 4 summarizes the core simulation and evaluation settings. The primary evaluator uses 60 steps at nominal 15 Hz, with exposure-dependent timing and earlier terminal events. Start, front wall, and goal are at local x=−1.5,0,1.5 m; the back wall is at x∈[2.00,2.85] m. The aperture has half-width 0.15 m, reset-sampled center y∼U[−0.75,0.75] m, and vertical parameter z=1.50 m. Random yaw rotates the complete scene; geometry is static within each episode. Glare affects the aperture halo, whereas dark material and specularity affect adjacent-wall returns (Figure 4).

Table 5 summarizes the stage-wise training settings.

Table 3 summarizes the two supervision collections. The initial teacher collection $D_{0}$ is configured with three regimes, four rollout batches per regime, 12 parallel trajectories per batch, and 60 steps per trajectory. The collector queries the teacher at every step and stores the full horizon without early termination, giving 3×4×12=144 sequences and 144×60=8640 state-wise labels (48 sequences and 2880 labels per regime). Each label contains the three camera registers. Student-rollout relabeling uses the same batch structure with 80 steps per trajectory, giving 144 sequences and 11,520 labels. These training-sequence horizons are independent of evaluation stopping limits. One relabeling round requires 11,520×120=1,382,400 state–optimizer-step evaluations, implemented as 115,200 batched updates of 12 states, excluding collection time.

### 4.2. Compared Policies

Table 6 separates complete-policy comparisons from intermediate training stages. Nominal fixed acquisition uses (P,E,G)=(0.5,0.5,0.5); random-fixed draws P∈[0.10,0.95], E∈[0.10,0.92], and G∈[0.02,0.90] once per episode. Non-diff tests a camera-learning procedure without differentiable sensor training; Blind tests dependence on depth. These references address the acquisition interface within the task. Photometric auto-exposure [6] requires image information absent from the rendered depth interface, and external navigation systems differ in observations and training. The physical D455 comparison supplies a separate device-auto-exposure reference. We claim improvement over the evaluated complete policies, rather than a universal navigation ranking.

### 4.3. Ablation Settings

Ablations use a 5 s timeout and 120-step cap, with three training runs evaluated on the same 900 episodes (300 per regime): 2700 executions per variant. Their 77.89% full result is not a replacement for the primary success of 75.1%. The stem comparison uses architecture-specific base models, 3000 base updates, 50 teacher steps, 120 epochs for each camera-fitting stage, and 3000 flight-adaptation updates. Shared stems remain trainable during flight adaptation; split stems preserve the camera encoder. Paired intervals use 10,000 hierarchical bootstrap resamples.

The no-DAgger comparison retains split stems and the common downstream adaptation budget, omitting only student-rollout relabeling and camera retraining. We report run means and sample standard deviations. Its 20,000-resample bootstrap is stratified by regime and resamples episode identifiers jointly across runs; intervals are conditional on the evaluated model pairs. Both ablations compare final navigation, rather than camera fit alone.

### 4.4. Metrics and Supplementary Robustness Protocols

Success requires reaching within 0.35 m of the goal at any evaluated step without collision over the rollout; collision overrides reaching. Collision uses radius-adjusted minimum clearance at the ten lookahead queries, checked before and after integration, with threshold 0.0011 m. Timeout denotes neither success nor collision. We average fill first over steps and then episodes, and report terminal goal distance over all outcomes. Primary binary intervals are Wilson 95% intervals; continuous metrics use episode bootstrap intervals, conditional on the policy.

Four added foreground layouts alter aperture visibility while retaining the frozen policy and primary evaluator. Layout selection uses separate validation seeds, 20 episodes per regime; testing uses 100 per regime for each layout and its paired original control (1500 episodes). Occlusion is the fraction of the initially visible aperture-observation ROI replaced by foreground returns, with wall-plane bounds $|y-y_{a}|<0.15$ m and |z−1.5|<0.75 m. Valid foreground depth can hide the opening without lowering global fill. Geometry and all regime outcomes appear in Appendix A.

The depth-bias test uses the same frozen split-stem policy, three bounds b∈{0,0.10,0.30} m, and 100 episodes per regime per bound (900 total). Episodes are paired across bias levels using identical scene randomization. An independently sampled 6×8 random field is bilinearly interpolated to 72×96 and held fixed in image coordinates within each episode. Its sign is selected once per episode, and its magnitude is 0.5b–*b* before clipping. Bias is added to every valid pixel before both policy branches; resulting depths are clipped to [0.05,6] m. Invalid pixels are unchanged for each individual rendering. Feedback may change subsequent trajectories and validity masks across runs. The smooth field defines a controlled systematic range perturbation. Paired success-change intervals use 10,000 regime-stratified bootstrap resamples.

### 4.5. Physical-Camera and Flight-Controller Experiments

The physical camera is an Intel RealSense D455 (Intel Corporation, Santa Clara, CA, USA), running firmware version 5.15.1.55. The D455 pilot uses one stationary setup and five blocks, each containing an 8 s capture from an adapted camera policy, device auto-exposure, and an illustrative manual 1 ms setting, with order permuted. Raw 640×480 depth is requested at 30 frames/s without postprocessing; full-frame coverage in [0.05,6] m is averaged by clip. Intervals resample the five clips or paired blocks, not correlated frames. A shared-stem camera branch was adapted by imitation of physically selected register targets; it differs from the frozen split-stem navigation policy. Appendix B gives the acquisition and adaptation settings.

In SIH, a physical open-source NxtPX4 flight-controller board (HKUST UAV Group, Hong Kong, China; https://github.com/Xiaodao-chen/NxtPX4 (accessed on 21 October 2026)) runs PX4 v1.15.4 dynamics/sensor simulation, estimation, and flight control. The hardware experiments use PyTorch 2.1.0 with CUDA 12.1. A host generates surrogate depth and runs the frozen policy, exchanging estimated state and acceleration/yaw commands through USB MAVLink. Rendering and outcome checks use SIH truth. The final comparison pairs 20 episodes per regime with pure simulation, sharing geometry, randomization, policy weights, and the 60-action limit. The final integration configuration is evaluated on fresh seeds following preliminary runs and is separate from the primary benchmark. No motors or physical vehicle motion are involved.

## 5. Results and Discussion

We first establish the navigation gain and the need for flight adaptation, then examine the learning design. The remaining tests assess acquisition behavior under occlusion, sensitivity to incorrect ranges, and execution through physical sensing and flight-controller interfaces.

### 5.1. Navigation Performance and Flight Adaptation

**Figure 5:**
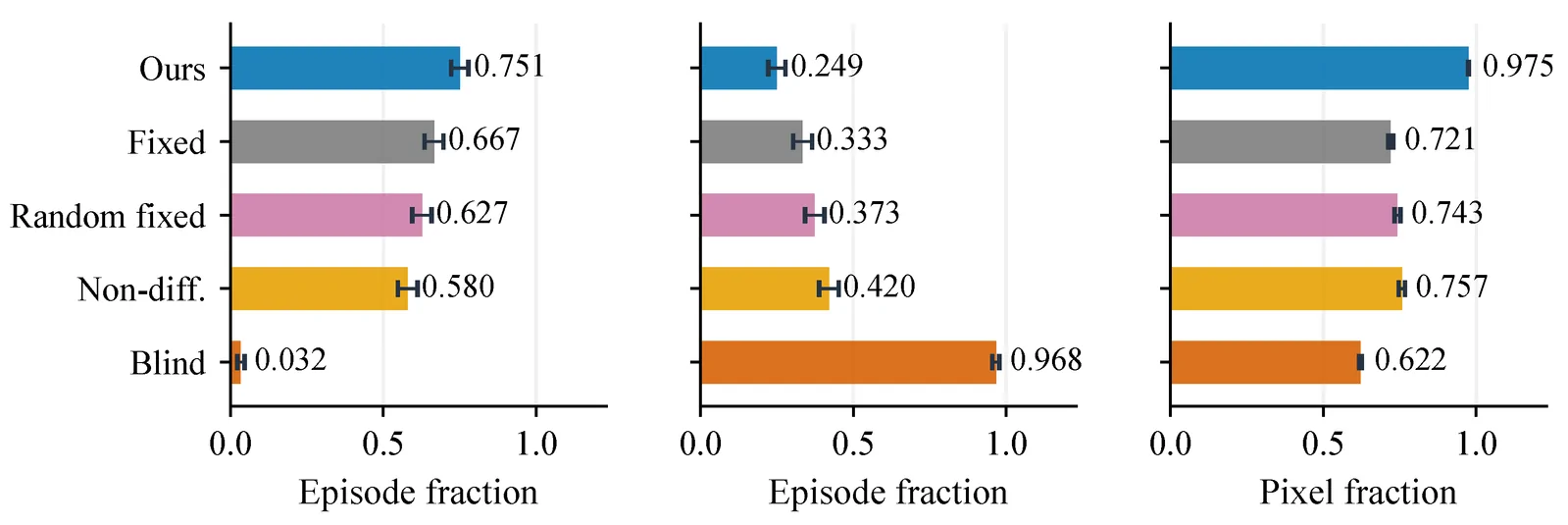
Held-out success, collision, and valid-depth fill rate. Fixed-camera denotes the nominal fixed-camera reference. Error bars show 95% confidence intervals: Wilson for binary outcomes and bootstrap for fill rate. For Blind, the plotted fill is a sensor-output diagnostic before input replacement; the depth input actually consumed by the policy is all zeros and has 0% valid-depth fill. The plotted value must not be interpreted as depth available to the blind controller.

The complete differentiable-acquisition and flight-adaptation framework improves navigation (Figure 5). Success is 75.1% versus 66.7% for nominal fixed, 62.7% for random-fixed, 58.0% for Non-diff, and 3.2% for Blind. Relative to nominal fixed, collision falls from 33.3% to 24.9%, fill rises from 72.1% to 97.5%, and mean terminal distance decreases from 0.672 to 0.550 m.

Success gains concentrate in dark material (68.3% to 82.0%) and specularity (66.3% to 76.3%). Glare fill increases from 68.7% to 95.8%, but success rises only from 65.3% to 67.0%. Global availability is, therefore, an imperfect proxy for crossing: the teacher improves weak patches without explicitly prioritizing aperture boundaries, and flight must exploit useful geometry in time. These results motivate learning control under the changed observation stream rather than equating high fill with navigation success.

**Figure 6:**
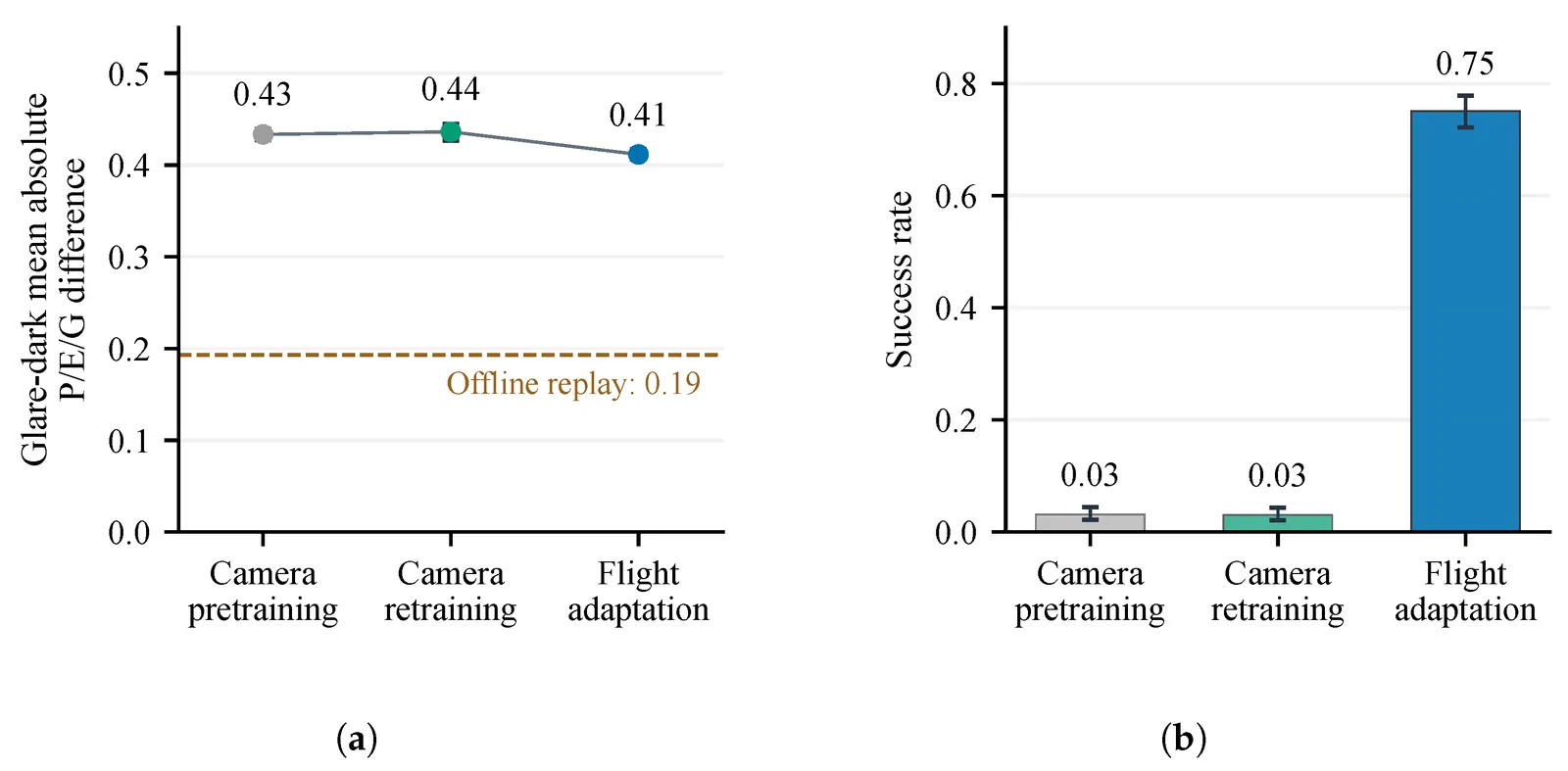
Camera-command separation (**a**) and success (**b**) after the three parameter-update stages. The dashed reference is the camera-retrained model’s target predictions on offline replay, not teacher labels. Points in (**a**) use rollout commands. Error bars are bootstrap 95% intervals for separation and Wilson 95% intervals for success.

The training-stage comparison makes that requirement explicit (Figure 6). The Pretrain and DAgger stages have approximately 95.8% fill but only 3.1% and 3.0% success. Flight adaptation raises success to 75.1% and fill to 97.5% while camera parameters remain frozen. Thus, reliable acquisition becomes useful through downstream adaptation; freezing its mapping still permits state-dependent register actions.

### 5.2. Ablation of the Learning Design

Under the separate ablation protocol, student-rollout relabeling plus camera retraining raises success by 7.11 points, from 70.78% to 77.89% (Table 7; paired 95% interval [5.78, 8.52]). All nine run–regime comparisons favor Full; across 2700 paired executions, 192 failures become successes and no success becomes failure. Fill changes only from 96.35% to 96.79%, while terminal distance decreases from 0.754 to 0.654 m. The navigation gain, therefore, exceeds what the small aggregate fill change alone would suggest. Full records 701/900 successes in each run, explaining its zero overall sample standard deviation despite different regime-wise rates.

Split stems improve overall success by 3.59 points over shared stems (77.89% versus 74.30%; interval [2.26, 4.89]), with fill increasing from 78.72% to 96.79% (Table 8). Glare is an exception: shared reaches 76.33% versus split 73.89%, with a split-minus-shared interval spanning zero. Preserving acquisition is beneficial overall, with regime-dependent gains. The comparison evaluates complete architectures, including their capacity and architecture-specific base models.

### 5.3. Acquisition Behavior and Partial-Observation Robustness

**Figure 7:**
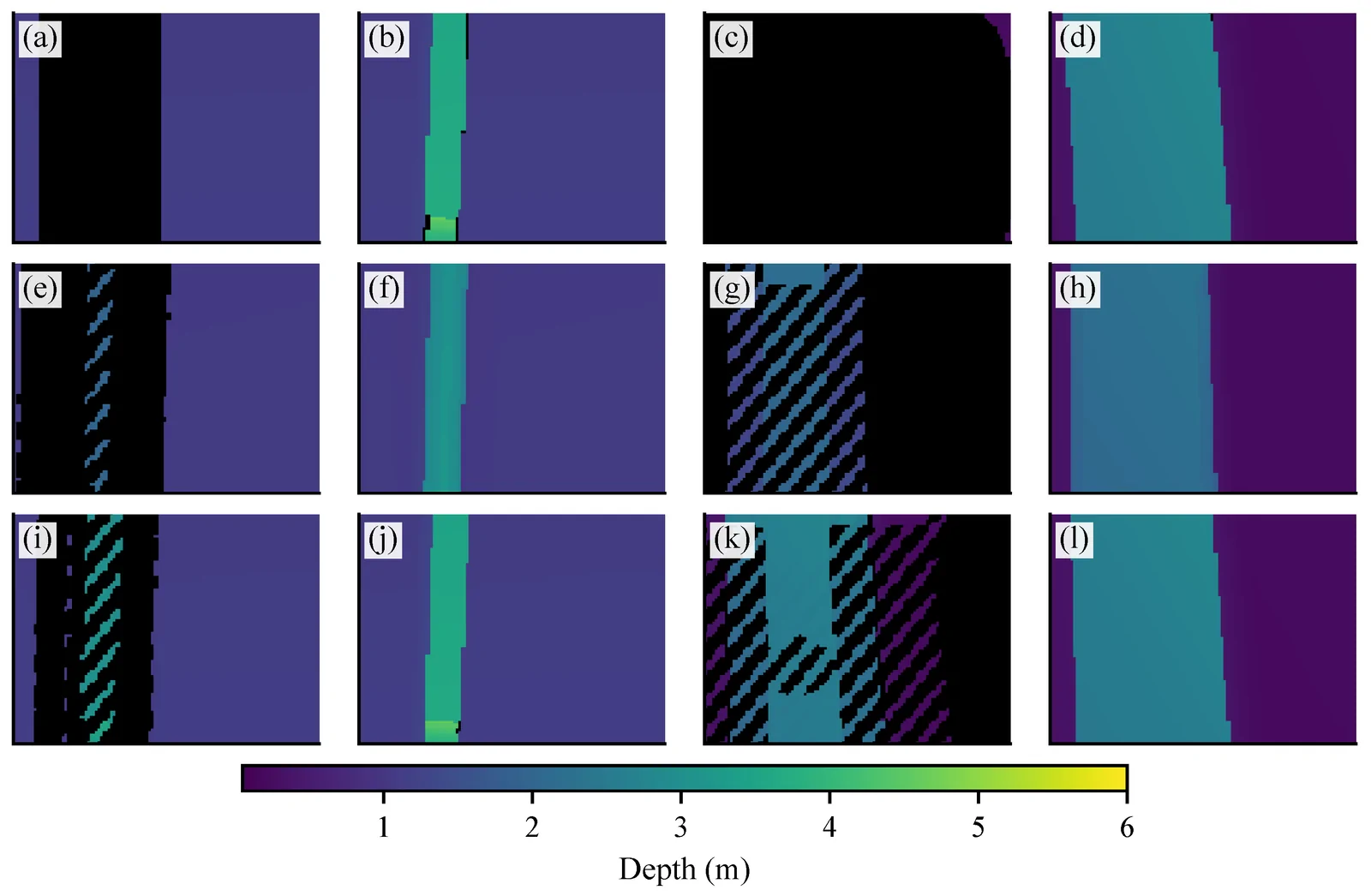
Matched-pose depth: (**a**–**d**) glare; (**e**–**h**) dark material; (**i**–**l**) specularity. Panels (**a**,**e**,**i**) and (**b**,**f**,**j**) show nominal fixed acquisition and Ours, respectively, at the first recorded pose; (**c**,**g**,**k**) and (**d**,**h**,**l**) show the same respective methods at the recorded pose nearest x=−0.30 m. Frames are selected by position, independently of image quality. Each pair shares geometry. The common depth scale is 0.05–6 m; black denotes invalid depth.

Figure 7 compares nominal fixed and learned acquisition at identical poses in all three regimes. The first recorded pose and the pose nearest x=−0.30 m are shown to compare acquisition early in the approach and near the opening. Near the aperture, mean P/E/G is 0.731/0.088/0.120 for glare, 0.665/0.694/0.683 for dark material, and 0.408/0.366/0.381 for specularity. These actions follow the surrogate’s response factors. Matched-pose replay isolates their observation effects; each policy’s closed-loop trajectories additionally reflect its visited states.

**Figure 8:**
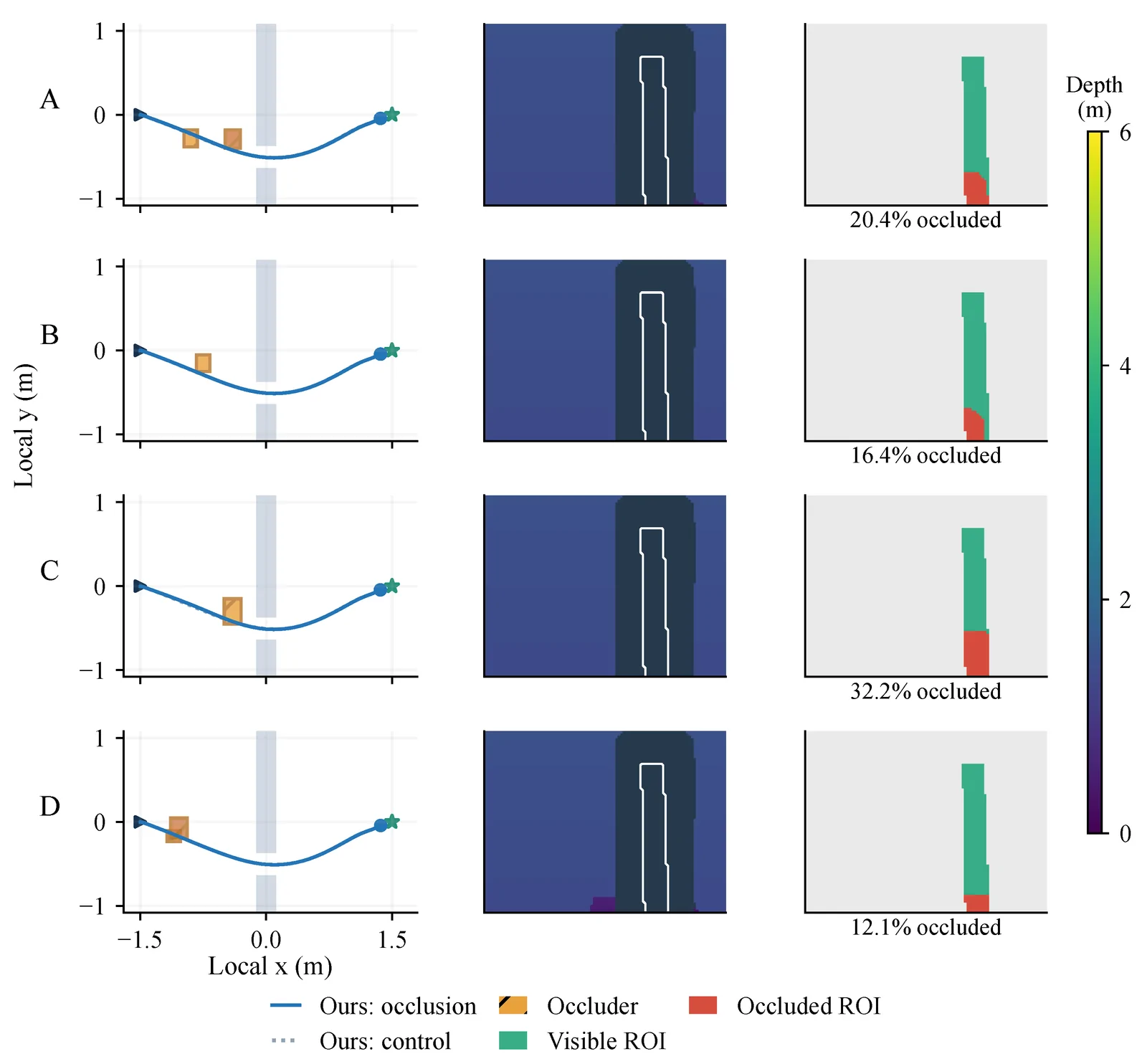
Four partial-observation layouts shown for the same preselected glare episode: (**A**) two staggered boxes; (**B**) one-sided box; (**C**) wider offset box; (**D**) stepped pair near the start. Left: Ours trajectories in the added layout (blue) and paired original scene (dotted gray); orange boxes are footprints, whose projected overlap need not imply collision. Middle: actual policy-input depth, with the original aperture-observation ROI outlined in white. Right: visible (green) and geometrically occluded (red) ROI pixels. Dark depth pixels are invalid measurements.

The four layouts vary how foreground geometry interrupts the approach. Layout A places two staggered boxes at different forward positions; B uses a one-sided box; C uses a wider offset box closer to the wall and D places a stepped pair near the start. The original wall, opening, and goal are retained. These boxes enter both depth rendering and collision queries, so the added geometry affects what the policy observes as well as where it can move. Figure 8 separates these effects: the trajectory panel shows the spatial response, the depth panel shows the acquired input, and the ROI panel identifies geometric occlusion. Unlike sensor dropout, geometric occlusion cannot be removed by changing acquisition registers. Navigation must, therefore, use the remaining observations as the viewpoint changes. The illustrated episodes are selected by identifier, while Table 9 summarizes the full evaluation set.

Added foreground occluders test whether the frozen policy retains this behavior when part of the opening is hidden. Across four validation-selected layouts, initial aperture-ROI occlusion is 11.97%–30.59%, yet success remains 75.33%–79.67%, versus 74.67% in the paired original control (Table 9). Figure 8 connects actual depth, visibility, and trajectories. This is retention of Ours, not a new baseline ranking; intervals do not establish improvement in every layout. Appendix A retains all conditions, including layout C’s dark-material decline from 83% to 78%.

### 5.4. Sensitivity to Biased Valid Depth

The bias experiment tests a failure mode absent from the training surrogate: valid measurements with incorrect ranges. The frozen policy retains 70.00% success at a 0.10 m bound, a 4.67-point decrease from its paired 74.67% zero-bias control. At 0.30 m, success falls to 37.00%, and collision rises to 63.00% (Table 10). Mean terminal distance increases from 0.562 to 0.633 and 1.138 m. All 900 episodes are included; no policy is retrained or selected using bias outcomes.

Fill remains 97.42% at 0.10 m and 96.08% at 0.30 m, despite the success loss. This directly demonstrates that availability cannot substitute for range correctness. The method retains substantial crossing capability under the smaller perturbation but is not robust to the larger systematic bias. The resulting failure boundary establishes range correctness as a separate requirement for reliable navigation: differentiable acquisition improves the availability of useful observations, while systematic range errors can still invalidate the geometry used by the controller.

### 5.5. Physical Acquisition and Hardware-in-the-Loop Navigation

**Figure 9:**
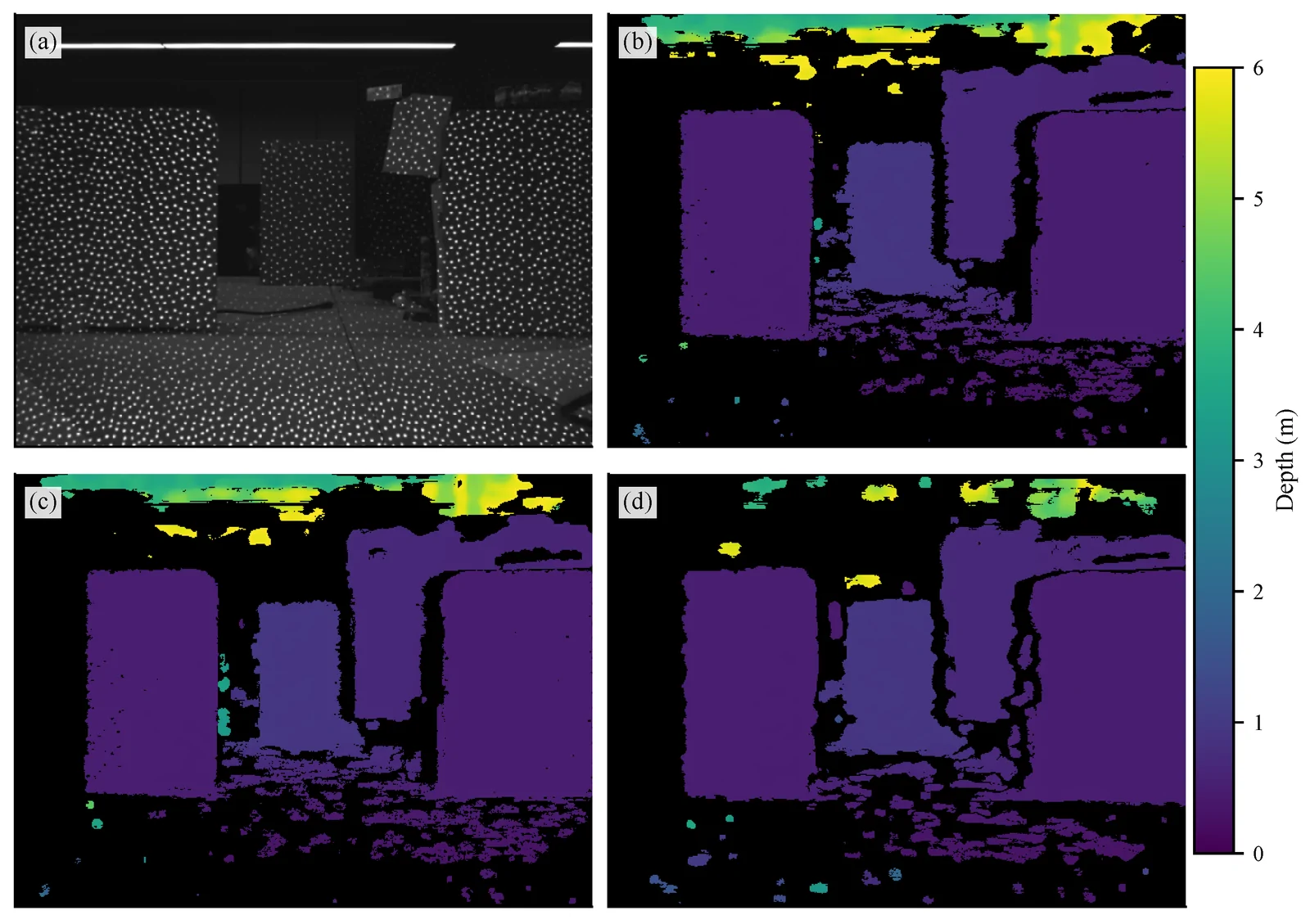
Physical D455 observations in the stationary setup: (**a**) left-infrared image at the adapted-policy capture; (**b**) adapted-policy depth; (**c**) device auto-exposure depth; (**d**) illustrative manual 1 ms depth. Frames are nearest the midpoint of each clip in the last complete acquisition block, rather than selected for maximum coverage. Depth panels use the same 0–6 m scale, with invalid/out-of-range pixels black and no hole filling. The views are sequential captures of the same stationary setup.

Physical acquisition improves after camera adaptation: D455 coverage is 50.07% versus 48.79% for device auto-exposure, a paired gain of 1.28 points (95% interval [1.21, 1.35]), positive in all five blocks. Throughput is 26.86 versus 29.99 frames/s (Table 11); the gain has a measurable delivery-rate cost. Figure 9 shows raw physical captures. The manual 1 ms setting was deliberately chosen as an underexposed illustration, not an optimized fixed baseline. This one-setup pilot establishes an executable camera interface after adaptation, rather than zero-shot surrogate transfer or depth accuracy.

**Figure 10:**
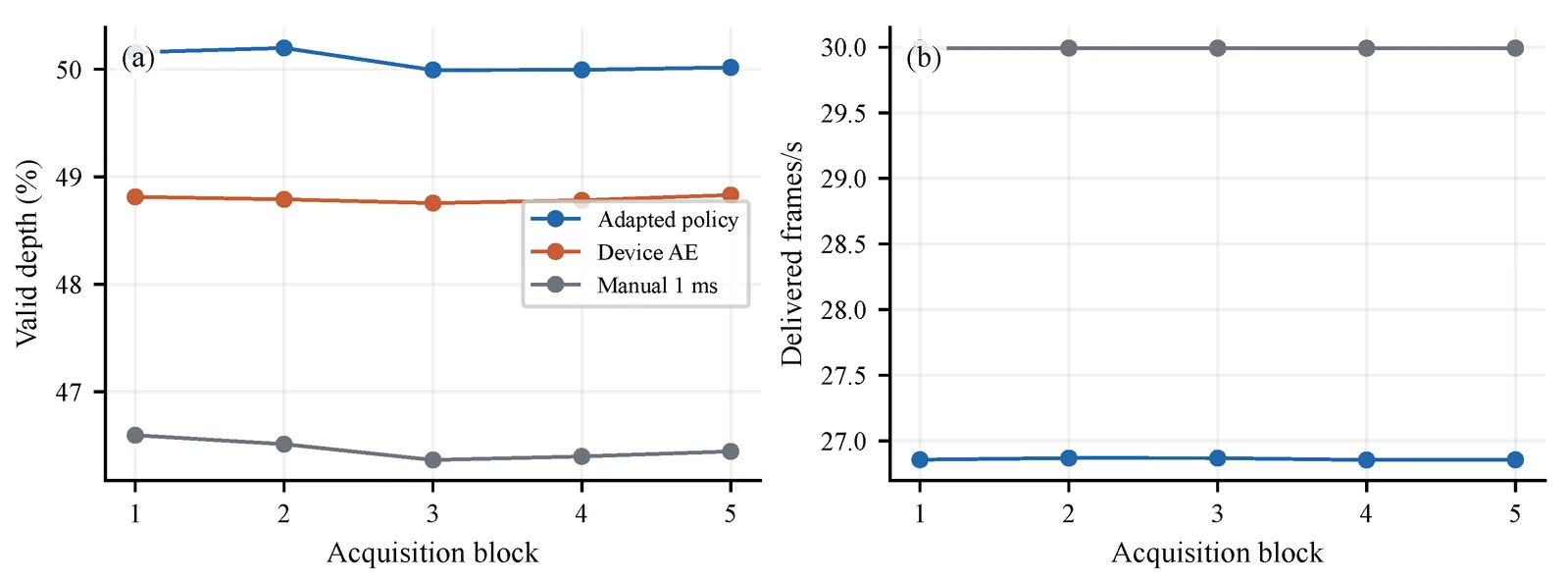
Physical D455 results by acquisition block: (**a**) full-frame valid-depth coverage and (**b**) delivered frame rate. Each point summarizes one 8 s clip; every block contains the adapted camera policy, device auto-exposure, and illustrative manual reference in permuted order. The two panels show the coverage gain and throughput cost within the same stationary setup.

Figure 10 shows the coverage–throughput tradeoff in all five blocks. Each point summarizes a complete clip; consistency across these repeated captures concerns one stationary setup, not independent scenes.

**Figure 11:**
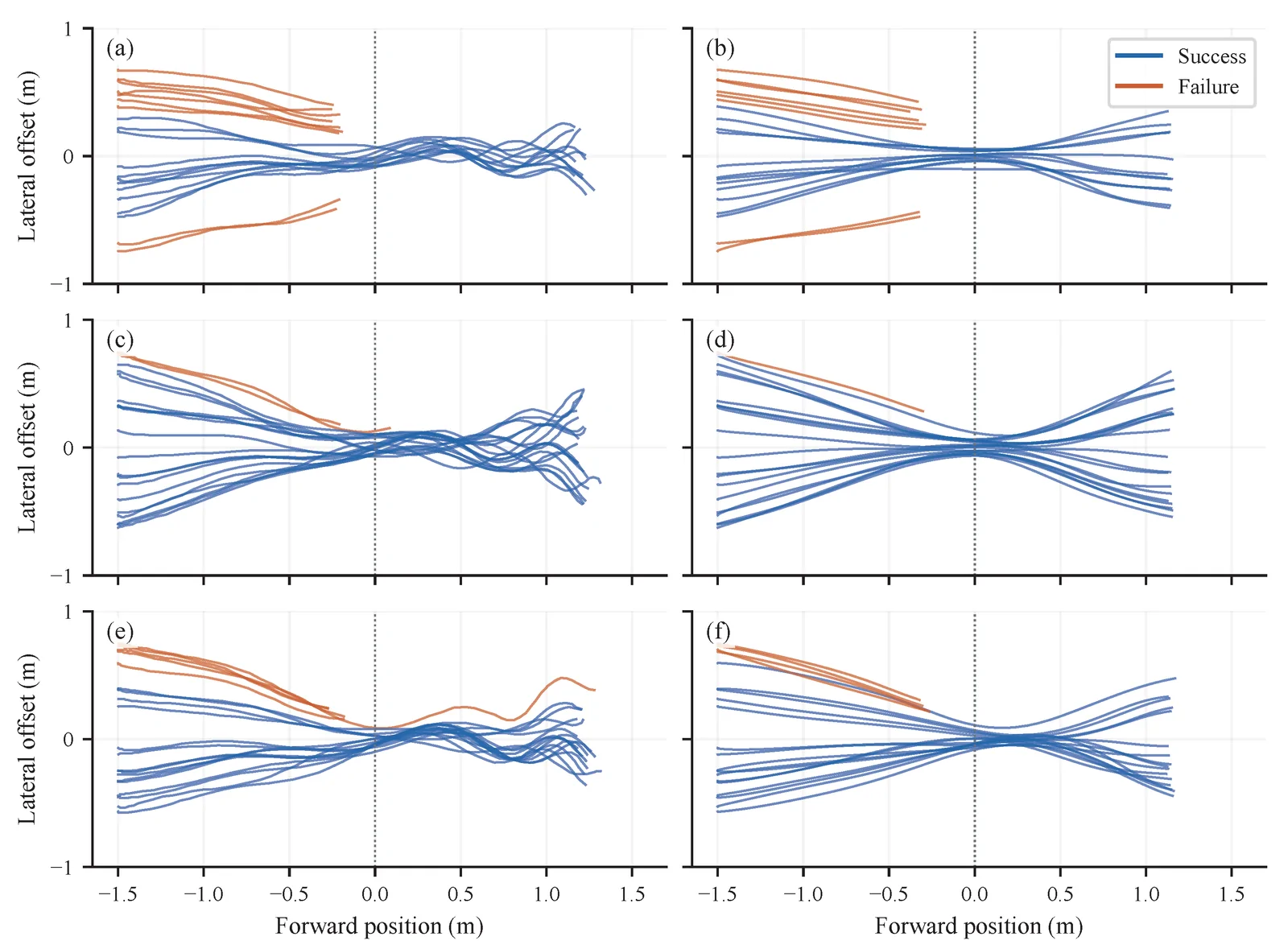
All 60 PX4 SIH trajectories (left column) and their 60 matched pure-simulation trajectories (right). Panels (**a**,**b**) show glare, (**c**,**d**) the specular proxy, and (**e**,**f**) the dark-material proxy; (**a**,**c**,**e**) are PX4 SIH and (**b**,**d**,**f**) are matched pure simulation. Each panel contains 20 episodes. Coordinates use the scene frame, with lateral position relative to the episode’s aperture center and the dotted line indicating the front-wall plane. Blue and orange distinguish successful and failed episodes. SIH traces use ground truth for evaluation; the policy acts on estimated state. The depth observations in both runs are synthetic.

The frozen navigation policy completes 44/60 SIH crossings (73.33%) versus 47/60 (78.33%) in matched pure simulation (Table 12). There are 44 joint successes, 13 joint failures, and three pure-simulation-only successes. All trajectories appear in Figure 11. The observed 5.00-point execution loss is descriptive, not an equivalence result. Across 2741 SIH steps, host processing, inference, and state-age 95th percentiles are 30.24, 8.63, and 20.91 ms; no scheduled slots are skipped. Per-trajectory scheduling spans 50.59–112.15 ms with jitter. Requiring 15 mm additional clearance retains 43/60 successes.

Together, the experiments connect differentiable acquisition to navigation gains and exercise physical sensing and flight-controller interfaces. The evidence concerns aperture crossing under the stated sensing and state-estimation models. D455 evaluates a separately adapted camera branch in a stationary setup; SIH evaluates the frozen navigation policy with synthetic depth, simulated dynamics, and hardware-based estimated-state feedback. These complementary tests establish acquisition feasibility and control-interface compatibility. The smaller glare gain and the severe-bias failure boundary delimit the navigation benefit: improved depth availability must be coupled with flight adaptation and sufficiently accurate ranges.

## 6. Conclusions

This study presents a staged learning framework that improves UAV navigation by coupling differentiable depth sensing with flight adaptation. Reliability-based supervision and student-state relabeling train the camera policy, while a separate flight branch adapts to the resulting observations without changing the learned camera mapping. In the primary aperture-crossing benchmark, success increases from 66.7% with nominal fixed acquisition to 75.1%. Ablation studies support the learning design, while additional layouts and depth-bias tests characterize performance under varied observation conditions. Physical D455 acquisition and PX4 SIH experiments provide complementary evidence on sensing and controller integration.

The evaluation focuses on aperture crossing under controlled sensing conditions, allowing the relationship between depth acquisition and navigation performance to be examined systematically. The findings, therefore, apply to the scene and sensor configurations studied, with broader applicability across environments, platforms, and operating conditions requiring further investigation.

Building on these findings, future work will extend the framework to more general navigation tasks and sensing platforms. This will involve learning from physical sensor data and integrating adaptive depth acquisition more closely with state estimation and flight control, with the aim of supporting navigation in complex environments.

## Figures and Tables

**Table 1:** Numerical targets and objective weights in the accompanying implementation. The parameter names follow the teacher and flight objectives in Section 3; a zero weight disables the corresponding penalty.

Parameter	Symbol	Value
Reliability target and patch selection		
Patch grid	$R_{p}\times C_{p}$	6×8
Lower-tail fraction	β	0.25 (12 patches)
Reliability target	$r_{\min}$	0.9
Nominal-recovery margin	$m_{\mathrm{fill}}$	0.25
Projector-power reference	$P_{0}$	0.5
Teacher objective weights		
Reliability shortfall	$\lambda_{f}$	50
Register-change penalty	$\lambda_{s}$	$5\times 10^{-3}$
Excess-power penalty	$\lambda_{P}$	0
Motion-dependent blur	$\lambda_{b}$	$1.5\times 10^{-4}$
Gain penalty	$\lambda_{G}$	$7\times 10^{-4}$
Nominal-register recovery	$\lambda_{0}$	0.075
Camera fitting		
SmoothL1 transition	δ	1
Temporal fitting penalty	$\lambda_{\Delta c}$	0
Flight objective weights		
Velocity tracking	$\lambda_{v}$	10.5
Acceleration regularization	$\lambda_{a}$	0.05
Jerk regularization	$\lambda_{j}$	0.05
Collision barrier	$\lambda_{c}$	6

**Table 2:** Training and evaluation settings that differ between the primary and ablation comparisons. Outcomes are compared within each protocol.

Quantity	Primary Comparison	Ablation Comparisons
Teacher updates per state	120	50
Camera pretraining/retraining	180/120 epochs	120/120 epochs
Evaluation stopping limits	60 steps	5 s timeout and 120-step cap
Episodes per regime	300 per method	300 per method per run
Reported aggregation	900 episodes per method	Three runs; 900 episodes per method per run

**Table 3:** Teacher-supervision collections. Each stage uses four rollout batches per regime and 12 parallel trajectories per batch. Steps per sequence denote stored training horizons, independently of evaluation limits.

Collection Stage	Sequences	Steps/Sequence	State Labels
Initial teacher collection $D_{0}$	144	60	8640
Student-rollout relabeling	144	80	11,520

**Table 4:** Core simulation and evaluation settings. Evaluation uses 300 episodes per sensing regime for each primary method.

Setting	Value
Control frequency	15 Hz
Evaluation horizon	60 steps
Rendered depth resolution	96×72
Policy depth resolution	48×36
Camera action	power/exposure/gain in ${\left[0,1\right]}^{3}$
Sensing regimes	glare, dark material, specular
Aperture center	y∼U[−0.75,0.75] m at each reset; z=1.50 m
Scene yaw	randomized
Evaluation episodes	900 per primary method

**Table 5:** Stage-wise training settings. Camera-fitting epochs follow the primary configuration reported in this study; optimizer settings and the flight-adaptation budget are specified from the accompanying training implementation. Each stage updates only its designated parameters.

Stage	Optimization	Budget and Batch Size
Camera teaching	Adam; learning rate 0.08	120 updates per state; 12 states per batch
Camera pretraining	AdamW; learning rate $10^{-3}$	180 epochs; 8 sequences per batch
Camera retraining	AdamW; learning rate $5\times 10^{-4}$	120 epochs; 8 sequences per batch
Flight adaptation	AdamW; learning rate $8\times 10^{-5}$, cosine decay to $8\times 10^{-7}$	3000 scheduled updates; 96 trajectories per batch

**Table 6:** Policies used in the quantitative comparison and diagnostic analysis. Only the first five methods are primary navigation baselines; pretrain and DAgger are included to separate camera semantics from flight adaptation. The no-DAgger and shared-stem variants are defined below.

Method	Role	Description
Ours	Main model	split-stem learned camera with flight adaptation
Nominal fixed	Baseline	nominal constant camera setting (fixed-camera in plots)
Random-fixed	Baseline	episode-constant random camera setting
Non-diff.	Baseline	learned camera without differentiable sensor training
Blind	Baseline	zero-depth controller
Pretrain	Diagnostic	camera-pretrained policy before student-rollout relabeling
DAgger	Diagnostic	camera-retrained policy before flight adaptation

**Table 7:** Student-rollout relabeling and camera-retraining ablation after flight adaptation. Success rates (↑) are means ± sample standard deviations across the training runs described in Section 4.3. Each variant has 2700 test executions, including 900 per regime. Differences are Full minus no-DAgger in percentage points (pp). Both use split stems and the same flight-adaptation budget.

Regime	No-DAgger (%)	Full (%)	Δ (pp)
Overall	70.78±1.02	77.89±0.00	+7.11
Dark material	74.22±0.51	81.67±0.88	+7.44
Specular	71.33±2.52	78.11±0.51	+6.78
Glare	66.78±1.26	73.89±0.51	+7.11

**Table 8:** Visual-stem comparison under the settings in Section 4.3. Success rates (↑) summarize 2700 paired test executions per variant, including 900 per regime. Differences are split minus shared in percentage points (pp). Intervals are paired hierarchical-bootstrap 95% intervals.

Regime	Shared (%)	Split (%)	Δ (pp)	95% Interval (pp)
Overall	74.30	77.89	+3.59	[2.26, 4.89]
Dark material	73.33	81.67	+8.33	[6.11, 10.67]
Specular	73.22	78.11	+4.89	[2.11, 7.44]
Glare	76.33	73.89	−2.44	[−5.78, 1.11]

**Table 9:** Frozen Ours: 300 episodes per setting (100 per regime). Occl.: mean initial aperture-ROI occlusion; Distance: mean final goal distance over all outcomes; ΔSR: paired change from the within-suite original control, in percentage points (pp). All timeout rates are 0%.

Layout	Occl. (%)	Success (%) ↑	Collision (%) ↓	Distance (m) ↓	ΔSR (pp)
Original	0.00	74.67	25.33	0.550	+0.00
A: staggered pair	19.19	79.67	20.33	0.472	+5.00
B: one-sided	14.50	76.33	23.67	0.523	+1.67
C: offset wide	30.59	79.00	21.00	0.480	+4.33
D: stepped	11.97	75.33	24.67	0.540	+0.67

**Table 10:** Frozen-policy bounded-depth-bias test: 300 paired episodes per setting, 100 per regime. Intervals describe paired success changes conditional on the policy and perturbation. All timeout rates are 0%.

Bias Bound	Success (%)	Collision (%)	Fill (%)	Δ Success [95% CI] (pp)
0 m	74.67	25.33	97.60	Reference
0.10 m	70.00	30.00	97.42	−4.67 [−8.00, −1.33]
0.30 m	37.00	63.00	96.08	−37.67 [−43.33, −32.33]

**Table 11:** Stationary D455 acquisition: five 8 s clips per method. Coverage is averaged per clip; brackets give 95% clip-bootstrap intervals. Frames/s is measured delivered throughput. The manual setting is an illustrative underexposed reference.

Acquisition	Frames	Valid Depth (%)	Frames/s
Adapted camera policy	1077	50.07 [50.00, 50.15]	26.86
Device auto-exposure	1200	48.79 [48.77, 48.82]	29.99
Manual 1 ms (illustrative)	1200	46.46 [46.39, 46.53]	29.99

**Table 12:** Matched pure-simulation and PX4 SIH navigation with the frozen policy. Each regime has 20 paired episodes with shared geometry and seeds. Counts retain all outcomes; these supplementary results are separate from the primary benchmark.

Sensing Regime	Successful Crossings		SIH Failures	
	Pure sim.	PX4 SIH	Collision	Timeout
Glare	12/20	11/20	9	0
Specular proxy	19/20	18/20	2	0
Dark-material proxy	16/20	15/20	4	1
All regimes	47/60	44/60	15	1
Success rate	78.33%	73.33%		

## Data Availability

The code, configuration files, evaluation scripts, and trained model weights are openly available in the public GitHub repository at https://github.com/Gaiwqqq/ActiveDepthSensingForUAV (accessed on 5 October 2026). The supporting data for the simulation, bounded-depth-bias, physical D455 acquisition, and PX4 SIH experiments will be made openly available in the same repository upon publication.

## Abbreviations

The following abbreviations are used in this manuscript:

DAgger	Dataset Aggregation
EMA	Exponential Moving Average
GRU	Gated Recurrent Unit
ROI	Region of Interest
UAV	Unmanned Aerial Vehicle
P/E/G	Projector power/exposure/gain
SIH	Simulation-in-Hardware

## Appendix A. Occlusion Layouts and Evaluation Protocol

The same frozen split-stem policy is evaluated across all layouts. Table A1 reports the condition-specific outcomes. Layout selection uses a separate validation set of 20 episodes per regime, ranked by Ours success, then baseline advantage, worst-regime success, and terminal distance. Testing uses 100 independent episodes per regime, paired with the original-scene control. Overall paired success changes for A–D are +5.00, +1.67, +4.33, and +0.67 percentage points, with 95% intervals [2.00, 8.00], [−1.00, 4.33], [0.00, 8.67], and [−1.00, 2.33]. Intervals use 10,000 paired regime-stratified bootstrap resamples, without multiple-comparison correction, and are conditional on the selected layouts.

**Table A1:** All condition-specific success rates (SR); 100 episodes per cell. Δ: change from the same-regime original control in percentage points (pp). The dark-material decrease for C is retained.

Layout	Glare		Specular Proxy		Dark-Material Proxy	
	SR (%)	Δ (pp)	SR (%)	Δ (pp)	SR (%)	Δ (pp)
Original	65.0	+0.0	76.0	+0.0	83.0	+0.0
A: staggered pair	73.0	+8.0	82.0	+6.0	84.0	+1.0
B: one-sided	67.0	+2.0	79.0	+3.0	83.0	+0.0
C: offset wide	75.0	+10.0	84.0	+8.0	78.0	-5.0
D: stepped	65.0	+0.0	77.0	+1.0	84.0	+1.0

Foreground boxes share scene yaw and remain fixed within an episode. Their lateral coordinates follow the reset-sampled aperture center $y_{a}$:

(A1) $$y_{o}=\alpha_{o}y_{a}+\delta_{o},\;\alpha_{o}=\frac{x_{o}-x_{\mathrm{start}}}{x_{\mathrm{wall}}-x_{\mathrm{start}}}=\frac{x_{o}+1.5}{1.5}.$$

The offset $\delta_{o}$ distinguishes the layouts. Table A2 gives all box centers and half-extents; the boxes participate in both depth rendering and the original collision queries.

**Table A2:** Local geometry of the additional occluders. Each box has center $\left(x_{o},\alpha_{o}y_{a}+\delta_{o},z_{o}\right)$ and the listed half-extents. Here $y_{a}$ is the reset-sampled aperture center. All boxes share the scene yaw; their geometry remains static within an episode.

Layout	Obj.	$x_{o}$ (m)	$\alpha_{o}$	$\delta_{o}$ (m)	$z_{o}$ (m)	Half-Extents $\left(h_{x},h_{y},h_{z}\right)$ (m)
A: staggered pair	1	−0.90	0.4000	−0.080	0.600	(0.080,0.100,0.600)
A: staggered pair	2	−0.40	0.7333	+0.080	0.575	(0.090,0.110,0.575)
B: one-sided	1	−0.75	0.5000	+0.100	0.625	(0.080,0.100,0.625)
C: offset wide	1	−0.40	0.7333	+0.070	0.625	(0.100,0.150,0.625)
D: stepped	1	−1.10	0.2667	−0.035	0.655	(0.080,0.065,0.655)
D: stepped	2	−1.04	0.3067	+0.080	0.635	(0.100,0.120,0.635)

## Appendix B. Supplementary Hardware Protocols

D455 optical-axis depth is projected to 96×72 using nearest-neighbor pinhole mapping, preserving invalid pixels. Motion inputs represent the stationary camera. A physical parameter search supplies the register targets for 300 camera-head imitation updates (Table A3). This adapted shared-stem camera model is separate from the frozen split-stem navigation policy used in SIH. The illustrative 1 ms manual reference is deliberately underexposed; device auto-exposure provides the substantive physical-acquisition comparison.

SIH and its paired pure-simulation evaluation share scene geometry, randomization, and policy weights. The integration configuration is selected in preliminary trials, then evaluated on 60 independent paired episodes. The 44/60 SIH success rate has a 95% Wilson interval [60.99, 82.87]%, conditional on this configuration. PX4 estimates motion from simulated inertial and navigation signals; rendering and outcome evaluation use simulator ground truth. An additional 15 mm clearance test assesses sensitivity to the resolution of this feedback. SIH involves neither physical vehicle motion nor physical depth-camera feedback.

**Table A3:** Reproducible settings of the two supplementary hardware experiments. Physical and virtual acquisition settings are separate interfaces.

Item	Setting
Physical D455 acquisition	
Camera input/capture	96×72 projected depth/640×480 raw depth
Policy register ranges	Power: 0–360 SDK units; exposure: 0.5–20 ms; gain: 16–64
Register updates	2 Hz; smoothing coefficient 0.7; power step 30
Camera adaptation	300 camera-head imitation updates; 48 training clips, 12 held-out imitation clips
Hardware teacher target	Power 360; exposure 5 ms; gain 16
Evaluation	Five blocks; three methods; 8 s per clip
PX4 SIH navigation	
Flight controller	Open-source NxtPX4 board; PX4 v1.15.4; onboard SIH dynamics and sensor simulation
Control interface	USB MAVLink; host acceleration and yaw setpoints; PX4 estimated-state feedback
Acquisition/policy	96×72 synthetic depth; frozen split-stem policy
Execution	60 actions, including the final interval; per-trajectory exposure-dependent scheduling
Host control adapter	Drag compensation; 50 ms state prediction
Outcome thresholds	Goal tolerance 0.35 m; clearance threshold 1.1 mm; additional 15 mm sensitivity check
Final evaluation	20 episodes per regime; 60 paired SIH/pure-simulation episodes

## Appendix C. Bounded-Bias Evaluation Details

The bias test uses the same frozen split-stem policy as Appendix A, selected using unperturbed validation before bias evaluation. Scene geometry is paired across bias levels, while perturbations are sampled independently of scene randomization. The perturbation affects all valid pixels and preserves invalid pixels within each frame. Policy weights remain fixed, and success, collision, and timeout are mutually exclusive outcomes. Table A4 reports every regime; the zero-bias condition serves as the matched reference for degradation under systematic range error.

**Table A4:** Condition-specific success rates (SR), 100 episodes per cell, and overall mean terminal distance over all outcomes. The 300-episode zero-bias control is distinct from the 900-episode primary benchmark.

Bias Bound	Glare SR (%)	Specular SR (%)	Dark SR (%)	Overall Distance (m)
0 m	61	80	83	0.562
0.10 m	62	74	74	0.633
0.30 m	36	41	34	1.138
